# Bonding of PMMA to silicon by femtosecond laser pulses

**DOI:** 10.1038/s41598-023-31969-y

**Published:** 2023-03-28

**Authors:** Filippo Maria Conte Capodacqua, Annalisa Volpe, Caterina Gaudiuso, Antonio Ancona

**Affiliations:** 1 Dipartimento Interateneo Di Fisica, Politecnico & Università Degli Studi di Bari, Bari, Italy; 2 Institute for Photonics and Nanotechnologies (IFN), National Research Council, Bari, Italy

**Keywords:** Materials science, Materials for devices, Biomedical engineering

## Abstract

Many devices and objects, from microelectronics to microfluidics, consist of parts made from dissimilar materials, such as different polymers, metals or semiconductors. Techniques for joining such hybrid micro-devices, generally, are based on gluing or thermal processes, which all present some drawbacks. For example, these methods are unable to control the size and shape of the bonded area, and present risks of deterioration and contamination of the substrates. Ultrashort laser bonding is a non-contact and flexible technique to precisely join similar and dissimilar materials, used both for joining polymers, and polymers to metallic substrates, but not yet for joining polymers to silicon. We report on direct transmission femtosecond laser bonding of poly(methyl methacrylate) (PMMA) and silicon. The laser process was performed by focusing ultrashort laser pulses at high repetition rate at the interface between the two materials through the PMMA upper layer. The PMMA-Si bond strength was evaluated as a function of different laser processing parameters. A simple, analytical, model was set up and used to determine the temperature of the PMMA during the bonding process. As a proof of concept, the femtosecond-laser bonding of a simple hybrid PMMA-Si microfluidic device has been successfully demonstrated through dynamic leakage tests.

## Introduction

The joining of dissimilar materials^1^, particularly semiconductors and polymers, is becoming increasingly important in the manufacturing of hybrid structures and components for engineering applications^2^, including automotive^3^, electrical, aerospace, and healthcare industries^4^. Several joining techniques, such as thermal bonding, adhesive bonding, arc bonding, soldering, riveting, screwing, and anodic bonding, have been used for realizing micro-optical, mechanical, electronic, and fluidic device^5,6^. However, these traditional joining methods generally have drawbacks such as premature aging, degassing, photo-bleaching and low accuracy^7^. Moreover, they can depend on the material chosen, and, exploiting glues and chemicals, they might be harmful to the environment.

Laser transmission bonding exploiting ultrashort laser sources has been recently demonstrated^8^. This technique is based on transmitting (hence the name) the laser beam through the bulk of the upper substrate transparent to the laser wavelength. The beam, focused on the contact interface of the two layers, is absorbed by the opaque substrate, causing highly localized heating and phase transition, which allow the materials to bond together. Due to localized nonlinear absorption processes, i.e., multiphoton, tunnel absorption and avalanche ionization^9^, which are facilitated by high peak intensities of ultrashort pulses within the focal volume, this technique allows bonding also between two transparent materials without any intermediate absorbing layer^10^.

Ultrashort laser micro-bonding can represent an effective choice also in case of an upper transparent and a bottom opaque material otherwise difficult to join due to large difference in optical and thermal properties (e.g., coefficients of thermal expansion). In particular, for the microelectronic industry, the bonding of transparent polymers and glasses to semiconductors, mainly silicon, is crucial for the prototyping and production of numerous devices, including microfluidic.

Tamaki et al.^11^ bonded a non-alkali-glass substrate to a silicon substrate, exploiting a femtosecond (fs)-laser system with a pulse duration of 947 fs, a repetition rate of 500 kHz and a wavelength of 1558 nm. At this wavelength, both substrates were transparent, thus nonlinear absorption process was involved in the focused spot to achieve heat accumulation, melting at the interface and resolidification, resulting in a joint strength of 3.74 MPa.

Similarly, glass-silicon bonding was also demonstrated by focusing a high repetition rate (700 kHz) ultrashort laser radiation with pulse duration of 350 fs and emitting at 1045 nm^12^. A modified region, presumably being a mixture of silicon and glass, was detected close to the interface of the bonding seam.

High spatial resolution (< 20 μm) and high throughput micro-bonding procedure ofglass-silicon was also demonstrated using a 20-ps-laser emitting at 1060 nm and with high repetition rates, i.e., up to 4 MHz^13^. The maximum shear strength of the weld joint attained in this study was 85 MPa. Thanks to such excellent mechanical strength and hermetic properties, this technique was suggested to be very promising for wafer level packaging in MEMS and sensors.

Recently, ultrafast laser welding of silicon to copper and the absence of a physical limit for semiconductor–metal ultrafast laser welding have been demonstrated^14^. Shear joining strength measurements reveal 2.2 MPa between the samples, thus holding a high potential for applications. The potential of nanosecond-laser welding of silicon to other semiconductors has been also studied, revealing a shear joining strengths of > 10 MPa for all processed configurations^15^.

Despite the plenty of papers published on bonding of silicon to other materials, exploiting fs-laser pulse, how to produce a high-strength joint between silicon and plastic still remains a challenge, due to the differential thermal expansion experienced by the two materials^16^. The interest in this hybrid junction stems from the huge potential of these two materials in many applications, in particular for microfluidics where laser approach could prevent any potential fluidic channel collapse^17^.

In this work, we investigate the feasibility of bonding a PMMA thin plate sandwiched onto a silicon substrate by focusing a femtosecond (fs)-laser beam at the interface between the two materials. The influence of repetition rate, pulse energy, scan speed and distance between consecutive scan lines on the bonding strength is studied. An analytical model, based on existing heat accumulation models, is set up and used to determine the temperature of the PMMA during the bonding and evaluate the plausibility of the mechanism proposed to explain the bonding process.

As a proof of concept, the developed fs-laser micro-bonding technique is applied to seal a fs-laser-fabricated microfluidic PMMA-Si hybrid device. The effective sealing of a previously laser fabricated microfluidic channel is demonstrated by leakage tests under controlled pressure. Indeed, due to its flexibility, micrometric precision, and not needing any chemical, ultrashort laser technology is well suitable for assembling of microfluidic devices, where contamination and clogging are the principal drawback of other techniques^18^. The main advantages of the proposed bonding technique are its flexibility, due to the use of a scan head, and the sustainability, being a green and chemical-free process, with no risk to clog cavities or contaminate samples^21^.

## Materials and methods

In this study, high-purity optical-quality Poly(methyl methacrylate) (PMMA) (Vistacryl CQ, Vista Optics Ltd, thickness 1 mm) plates and silicon Si (100) wafers (type P doped with B, thickness 500 µm) have been used as material substrates. PMMA and silicon have been purchased with λ∕4 flatness and surface roughness < 5 nm. All the samples have been fs-laser cut to obtain dimensions of 30 mm × 30 mm. Prior to bonding, the Si samples were wiped with isopropyl alcohol and PMMA was sonicated in distilled water for 5 min to remove any processing debris.

For bonding, a Yb-doped disk ultrashort laser source (TruMicro 5050 Femto Edition, Trumpf GmbH) was used, emitting 900-fs pulses at 1030 nm and providing a linearly polarized almost diffraction limited beam (M^2^ < 1.3), at a maximum repetition rate of 800 kHz. The laser beam was handled by a galvoscanner (IntelliSCANse 14, SCAN-LAB) equipped with a telecentric lens of 100-mm focal length (LINOS F-Theta-Ronar 100 mm telecentric, Qioptiq Photonics GmbH). The focused beam had a spot size of about 30 µm^21^. The bonding seams were generated focusing the beam at the interface between the two substrates, pressed together by clamping arms, and moving the focal spot following a scan path consisting of adjacent lines. A good contact between the substrates was ensured by tightening the clamps until iridescence and interference fringes were observed. The focus position has been chosen after some preliminary tests performed on the silicon-polymer sandwich. Several scan lines were produced in different depths with respect to the interface and the focus position was chosen in which the most continuous modification of both materials was obtained.

**Figure 1:**
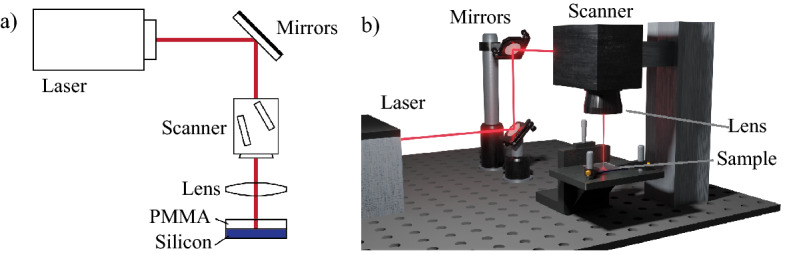
(**a**) Sketch and (**b**) 3D render of the laser bonding setup.

The optical chain from the laser to the sample in lap-join configuration is shown in Fig. 1a,b.

The laser parameters investigated for the laser bonding experiments are listed in Table 1. The range of investigated pulse energies was selected observing that below 1 μJ the substrates were unaffected by the laser beam, while exceeding 2.5 μJ extensive collateral damage to the surroundings of the irradiated area was present. The scanning speed values were chosen so that bonding areas of a few cm^2^ would require a processing time of the order of minutes.

**Table 1:** Laser parameters for PMMA-Si micro-bonding.

Parameter	Value
Pulse energy *E*_*p*_ (μJ)	1; 1.25; 1.5; 1.75; 2; 2.25; 2.5
Repetition rate *f* (kHz)	200; 400; 800
Scanning speed (mm/s)	0.01; 0.05; 0.1; 0.5; 1.0
Hatch distance *h* (μm)	19; 38; 56; 75; 110; 150

After laser irradiation, the laser-induced modifications on the substrates and the bonding area were analyzed through optical microscopy (Eclipse ME600, Nikon).

**Figure 2:**
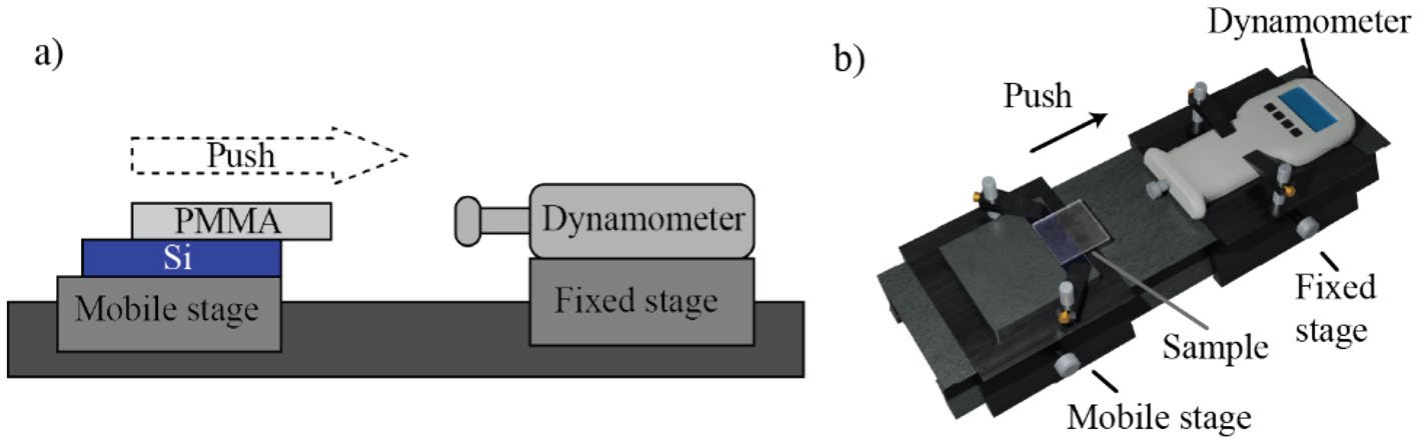
(**a**) Sketch and (**b**) 3D render of the setup built to measure the bond shear strength.

The bonding strength test were carried out in push-test configuration, as shown in Fig. 2a,b. The PMMA-Si laser-bonded samples were locked to a mobile translation stage, which was manually moved towards a dynamometer (FK 250, Fr. Sauter AG). It automatically recognized the maximum force applied (F) until the detachment. The bonding seam shear strength (S) was calculated as the ratio between F and the welded area (A): $S=F/A$. The bonding area A was directly measured using the optical microscope.

After the strength test, the silicon substrates, being the PMMA removed, were observed under a scanning electron microscope (SEM, Sigma 560 VP, Zeiss), and their bonded region was compared with a pristine region of the same sample. The silicon samples were then subjected to an electron dispersive X-ray (EDX) analysis using the same microscope.

**Figure 3:**
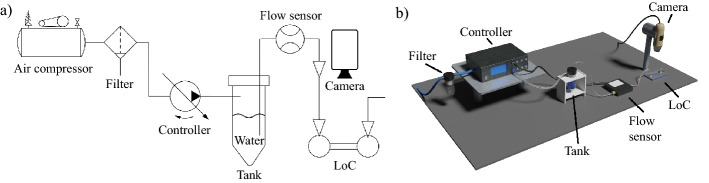
(**a**) Schematic representation and (**b**) 3D render of the setup built to test the LoC device.

Once the bonding technique was developed, it was used to assembly a hybrid simple Lab-on-a-Chip (LoC) device. It was constituted by a microchannel connecting two reservoirs, which represents the fundamental building block of any microfluidic device and were micro-machined in the PMMA substrate with the same laser source used for the bonding process. Additional information about the micro-machining process can be found in section "Proof of concept: microfluidic channel sealing and testing". To test the functionality of this device, a setup was built to pump distilled water through the channel at increasing pressures. Such apparatus, sketched in Fig. 3a,b, comprised a two-channel controller (OB1 MK3+, Elveflow), operating between 0.01 mbar and 2 bar, which used filtered compressed air to push distilled water from a tank into the tested device. Through its path, the water also flowed through a thermal time-of-flight flow rate sensor (Microfluidic Flow Rate Sensor, Elveflow). During the test, a camera (AD7013MTL, 20-90X magnification, Dino-Lite) was placed on top of the LoC to closely observe in real time the flow of water through the channel. Distilled water was injected into the microfluidic channel at increasing pressures, from 100 mbar to 1 bar with 100 mbar steps. Each pressure value was maintained for 1 min while the sealing was analyzed under the Dino-lite portable microscope to spot the precise moment in which the bond started to leak because of the water pressure.

## Results and discussion

Laser processing involves several laser parameters, e.g., pulse energy, polarization, pulse duration, and repetition rate. In this study, we focused on the influence of three of these, while keeping the others fixed. The three chosen parameters are the repetition rate andpulse energy of the laser beam, and the speed at which the beam scans the interface. These are the laser parameters that govern the laser energy released onto the workpiece per unit area and time together with the hatching geometry, defined here as the distance between consecutive scan lines, which was also considered and studied as a processing parameter.

## Influence of pulse energy and scanning speed

The influence of pulse energy and speed was studied at a fixed repetition rate of 800 kHz. Different combinations of these parameters were investigated by scanning single lines of length 1 mm for each setting and evaluating the quality of the bond by optical microscopy.

**Figure 4:**
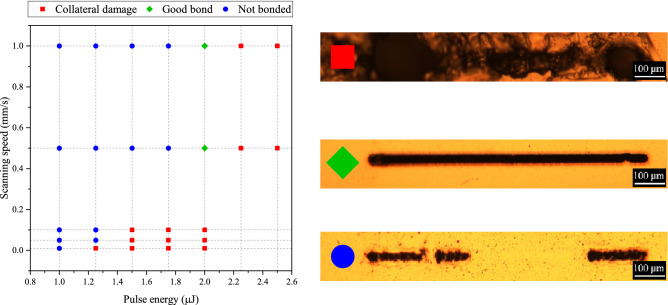
Single line bonding at different pulse energies and scanning speeds with repetition rate of 800 kHz. Each marker corresponds to a specific pulse energy-speed combination and its shape and colour indicates if bonding was achieved and if collateral damage was observed. An additional legend with pictures corresponding to each type of marker is shown on the right-hand side. Best results are obtained at 2.0 μJ,0.5 mm/s and 1.0 mm/s scanning speeds.

The range of pulse energies investigated was selected between 1 μJ and 2.5 μJ; values outside this range were not tested since at these two extremes negative results were already being observed. The scanning speed was studied up to a maximum value of 1 mm/s, since the target bonding dimensions are in the millimeter range and reaching higher speeds in such short distances would require short speeding ramps, leading to uneven energy delivery to the substrate and non-uniform bonding. Each point in Fig. 4 identifies a certain combination of pulse energy and speed. Blue dots indicate a combination for which a uniform bond was not achieved, while green diamonds identify combinations that resulted in a continuous and uniform bonding line. Finally, red squares represent combinations that generated extensive collateral damage in the substrate beyond the laser irradiated area, i.e. the PMMA melted completely over a large area and lost its optical cleanness.

It is found that, at a fixed repetition rate of 800 kHz, a suitable combination of pulse energy and travel speed is required to achieve a continuous and uniform joining seam^19,20^. At pulse energies less than 1.8 µJ, given the number of pulses per spot determined by the scanning speed used, the total heat input is not enough to melt the PMMA, so generating a non-continuous modified line. However, increasing the energy above 2.2 µJ, an uncontrollable damage of both the substrates compromises the bond homogeneity. A controlled and continuous joining line is achieved only in a narrow window of process parameters, namely at 2.0 µJ of pulse energy and 0.5 mm/s or 1 mm/s of translation speed. Between the two speeds, the latter was preferred for the next tests, since it allows to halve the laser processing times. To generalize this result, the deposited energy dose for the two best pulse energy-speed combinations is determined. This value is 6.7 kJ/cm^2^ for the combination 2.0 μJ, 1 mm/s, and 13.4 kJ/cm^2^ for the other one (2.0 μJ, 0.5 mm/s).

It Is worth noting that the selected combination of laser parameters (namely 2.0 μJ, 1 mm/s) was unable to modify the PMMA alone, neither if focalized into the bulk nor on the surface. This last result is consistent with what is already known in literature, since the fluence value used is below 3.2 J/cm^2^, which is the average fluence threshold for laser ablation of PMMA^21^; it can be therefore deduced that the presence of the silicon substrate is necessary to melt the PMMA and allow the bonding process. This hypothesis is discussed in section "Thermodynamic analytical model of the micro-bonding process".

## Influence of hatch distance

**Figure 5:**
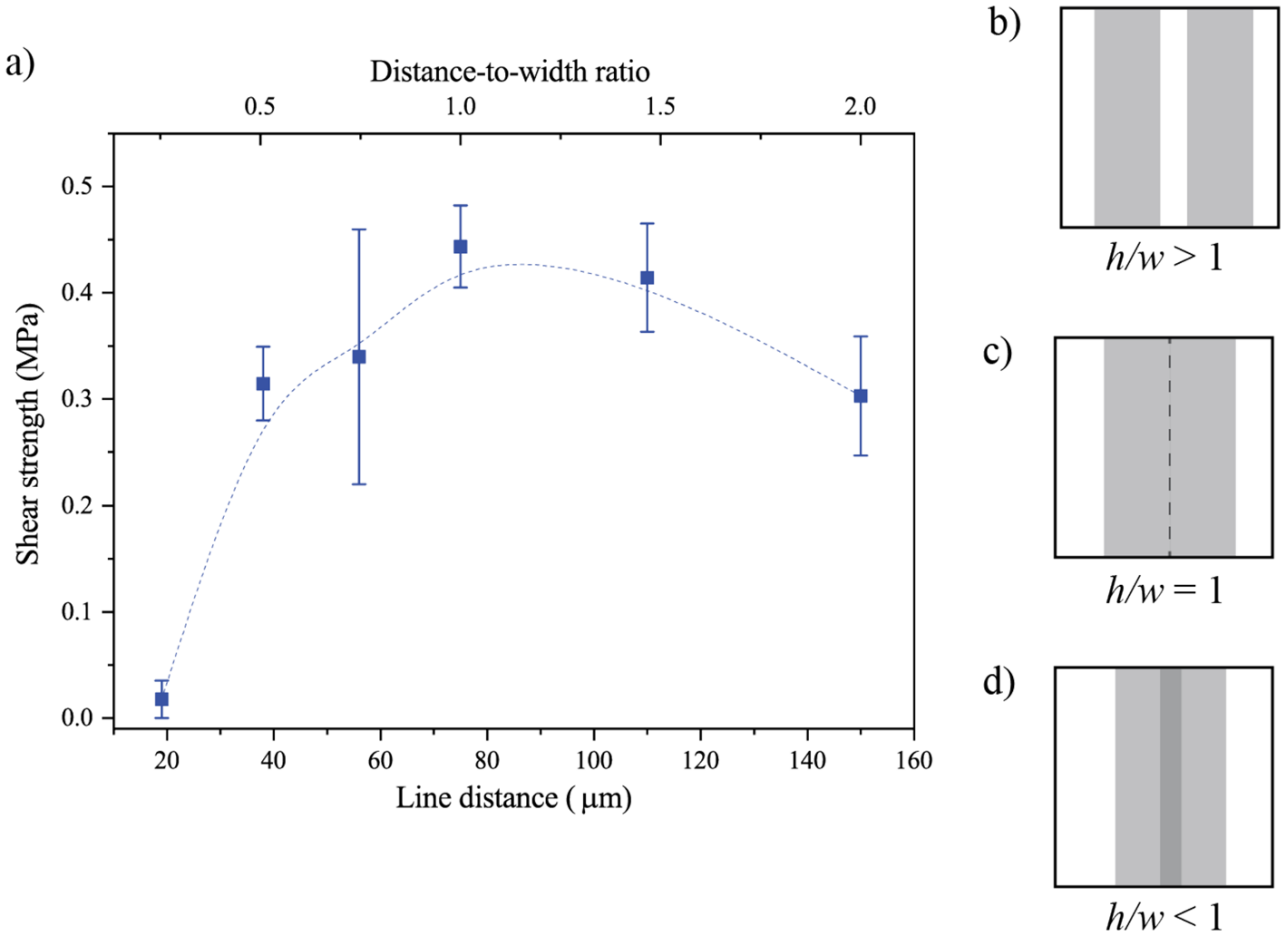
(**a**) Shear strength (with dashed trend line) of bonded samples as a function of the hatch distance at 2.0 μJ and 1 mm/s, and (**b**–**d**) sketches of the overlap between consecutive bonded lines for different values of h/w. The higher shear strength is obtained when h/w = 1.

The influence of the hatch distance between parallel scan lines on the shear strength of the bonded samples has been investigated. The laser parameters were kept fix (pulse energy 2 µJ, scan speed 1 mm/s), while the hatching distance *h* was varied in a range from a few tens of microns to over one hundred. A total of 50 parallel lines of 1 mm length were written for each hatching distance. In Fig. 5a, the measured shear strength is reported as a function of *h* and *h/w*, with *w* being the bonded line width (75 µm).

As shown in Fig. 5b, if *h/w* > 1 each line is well separated from the next one, if *h/w* = 1 (Fig. 5c) the lines are right one next to each other, and if *h/w* < 1 (Fig. 5d)) the lines overlap.

The maximum value of shear stress (i.e., 0.44 ± 0.04 MPa, for a written area of 3.75 mm^2^) has been observed when *h* is almost equal to the width of the bonding line, namely when *h/w* = 1. This result is particularly interesting since from literature a higher shear strength was expected for lower hatch distances. Indeed, in previous studies on fs-laser bonding^22^ of two PMMA layers it was found that defects and voids produced by each laser passage were filled with the molten material generated by the next laser line. Thus, increasing the overlap between consecutive laser line, resulted in an increase of the weld strength. Conversely, in our case, when the laser beam passes over a previously produced laser-modified line, i.e., when *h/w* < 1, a decrease in the shear strength can be noticed, despite no notable difference was observed under optical microscope between the bonded region of samples with h/w < 1 and the bonded region of samples with h/w = 1. This result can be ascribed to the “breaks” of the anchoring between PMMA and silicon, due to the scan of the laser on an already machined line. On the other hand, increasing the hatch distance has a negative, but less pronounced, impact on the shear stress; this could lead to the hypothesis that nearby lines have a reinforcing effect on each other.

As for the value of shear strength obtained (0.44 ± 0.04 MPa), it can be noted that it is lower than what is reported for the bonding of silicon with other materials, e.g., copper (2.2 MPa^14^) and glass (3.74 MPa^11^). This difference can be ascribed to the fact that copper and glass are more “similar” to silicon than PMMA is. For example, glass, which is mainly composed of silicon itself, bonds better to silicon than PMMA does, because of the similarity in composition between the two materials. Nonetheless, it should also be observed that the achieved shear strength is sufficient to ensure the functionality of the tested LoC device, as will be shown in section "Proof of concept: microfluidic channel sealing and testing".

## Influence of repetition rate

The influence of the repetition rate on the PMMA-Si bonding was also evaluated. Several tests were performed at three values of repetition rate (200, 400 and 800 kHz), keeping a fixed the number of pulses per spot of 24,000. The average power was changed to maintain a pulse energy of 2 μJ; the scanning speed was also adjusted in order to keep fixed the pulses per spot.

A number of 26 consecutive lines were scanned with a spacing of 75 µm, which is about the measured dimension of the laser track on silicon, employing the three different levels of repetition rate and keeping *h* to the value at which the maximum shear strength was achieved. The total joined area was about 1.8 mm^2^, which is a reasonable size for applications in micromachining of miniaturized devices, like e.g., bonding of micro-fluidic channels.

The two materials resulted bonded only at the repetition rate of 800 kHz. Conversely, at the other two repetition frequencies the two layers remained detached.

At 800 kHz, we can assume that heat accumulation process generates an increase of the PMMA temperature above the glass transition temperature (110 °C)^23^. Consequently, similarly to what observed by Miyamoto et al. for bonding of glass and silicon^13^, the softened polymer can flow into the rough laser ablated silicon, generating an anchoring.

**Figure 6:**
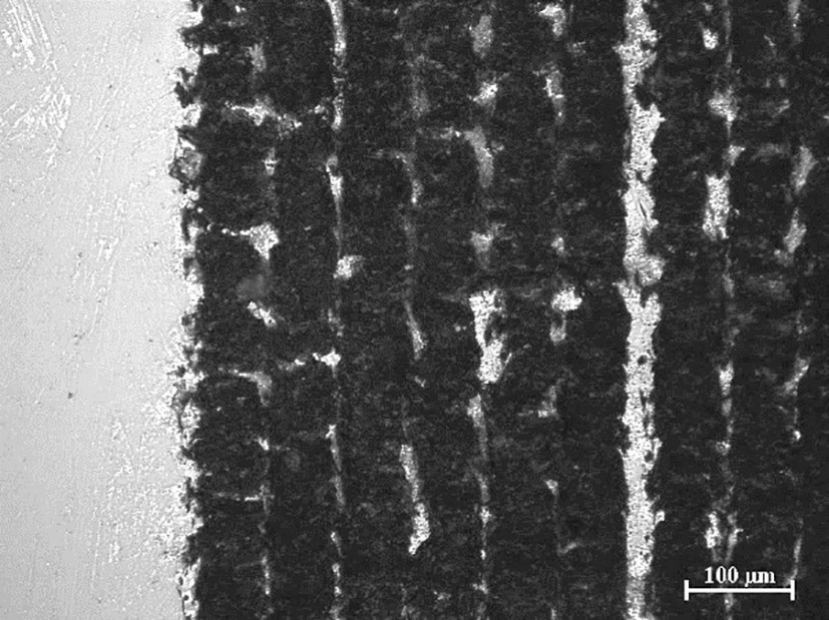
Optical image of the silicon substrate after laser bonding, and being the PMMA removed (pulse energy 2.0 μJ with repetition rate 800 kHz, scan speed 1 mm/s).

In Fig. 6, an optical microscope image of the silicon substrate surface after removing it from the bonded PMMA layer is shown.

A continuous and rough laser trace was detected, enabling anchoring of the melted PMMA. This mechanical interlocking mechanisms was deeply investigated by Xu et al.^24^ while trying to join plastics to stainless steel. Here, the metallic substrate was previously laser-textured and, then, heated and pressed against a plastic substrate. As a result, the thin plastic layer directly in contact with the hot metal melted and penetrated the micro-structure of the metal surface, producing micro-rivet mechanical joints. In our case, the fs-laser produced at the same time the texture on the silicon layer and the melting of the PMMA, resulting in the same joining mechanism observed in^24^.

**Figure 7:**
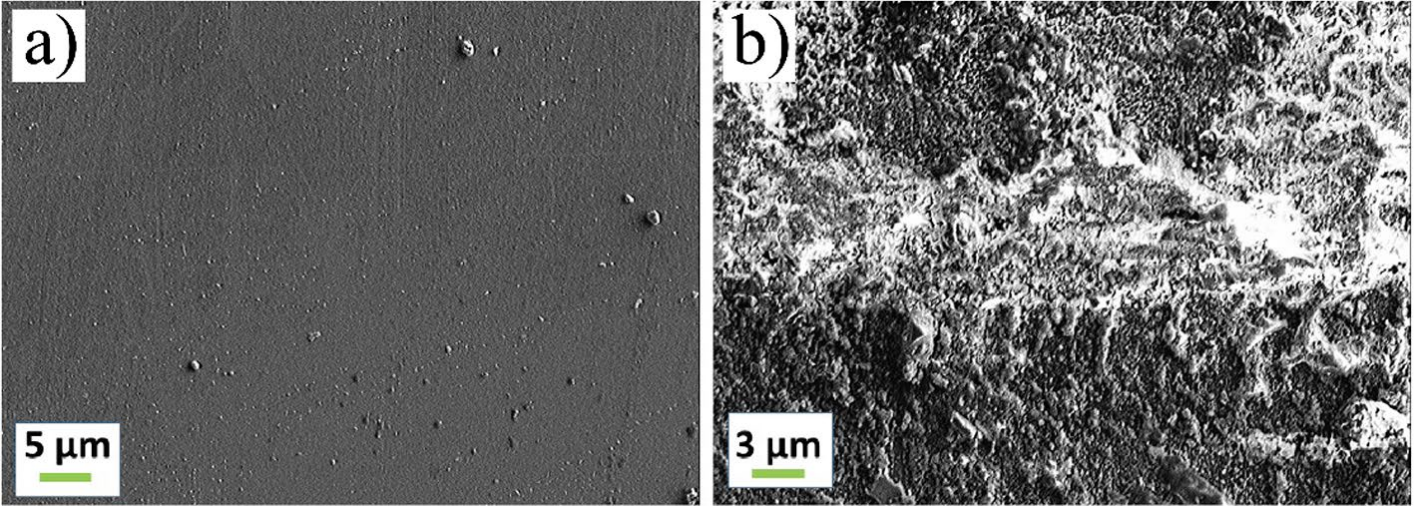
SEM images of a silicon wafer after bonding and being the PMMA removed. (**a**) Untextured area. (**b**) Bonding area at optimum laser parameters (pulse energy 2.0 μJ with repetition rate 800 kHz, scan speed 1 mm/s).

The proposed bonding mechanism is also supported by the observation under SEM, and the correspondent EDX analysis. of a silicon wafer after bonding and being the PMMA removed. In particular, the untextured area (Fig. 7a) is compared to the SEM image of the same silicon substrate in the machined area where the bonding occurs (Fig. 7b).

It can be observed that the passage of the laser generated an increase in roughness, where the melted PMMA can easily flow. The presence of anchored PMMA is proved by the different percentage of carbon on the surface. By the EDX analysis of the sample, a carbon percentage < 6% in the non-textured area emerges, compared to a carbon content > 20% in the bonded part. The non-zero percentage of C also in case of pristine sample can be ascribed to the PMMA debris evaporated during the laser process and remained attached to the silicon surface.

## Thermodynamic analytical model of the micro-bonding process

In the previous sections, it was shown that a focused beam with the following combination of laser parameters, i.e., 2.0 μJ pulse energy with 800 kHz repetition rate and 1 mm/s scanning speed, ensured on the one hand a uniform and continuous bonding at the interface between the two materials but, on the other hand it was unable to modify the bulk PMMA. Moreover, among all the investigated values of repetition rate, 200, 400, and 800 kHz, and keeping fix the pulse per spot, bonding between PMMA and silicon was obtained only at the higher frequency value.

These results can be explained assuming that to achieve the bonding, the PMMA need to be heated beyond its glass transition temperature and this transformation can take place only in a regime of heat accumulation, namely at relatively high repetition rates. In this case, the melted PMMA can flow into the trenches ablated into the silicon and then cool down, thus forming mechanical interlocks at the interface between the two materials. This hypothesis should be reasoned in terms of the ablation thresholds difference between the two materials, which is substantial: the silicon ablation threshold is about ~ 0.4 J/cm^2^^25^, whereas PMMA is 3.2 J/cm^2^. So, modification happens before in Si and then in PMMA, as proved by the fact that irradiating the sole PMMA with that combination of laser parameters does not produce melting. The only explanation to justify bonding between the two materials is that a relevant part of the energy needed to melt the PMMA is provided by heat transfer from the silicon substrate. A thermodynamic model of the process was thus developed to check the plausibility of this assumption.

The bond, as previously explained, was achieved by scanning a series of lines; each line, in turn, was composed by individual spots. For this reason, the study of the heating of the materials during the laser-matter interaction was first simplified to the study of a single irradiated spot. During each laser pulse, it was assumed that a fraction of the incoming radiation of intensity was absorbed by the PMMA, whose temperature increased of a quantity *ΔT*_*rad*_. The remaining part of the radiation was then transmitted through the PMMA and absorbed by the silicon, which heated up. Since the two layers were clamped together, it is likely that the silicon substrate transferred back to the PMMA part of this heat via thermal conduction. Thus, the PMMA temperature increased of an additional quantity *ΔT*_*cond*_.

**Figure 8:**
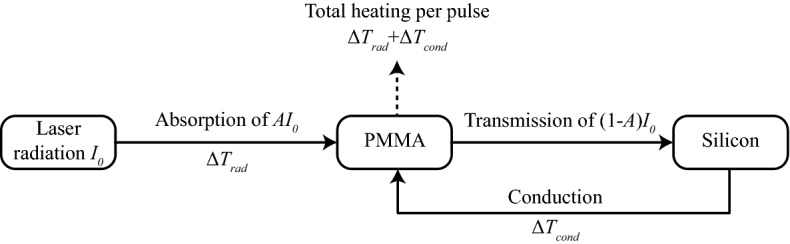
Schematic representation of the heating processes of PMMA during the irradiation by a single laser pulse.

A schematic representation of these absorption and conduction processes is shown in Fig. 8.

The PMMA temperature of the laser irradiated spot after delivering a number *N*_*p*_ of pulses per spot can be expressed as:

1 $${T}_{PMMA}={T}_{a}+\left(\Delta {T}_{rad}+\Delta {T}_{cond}\right)\cdot {N}_{p}$$

where *T*_*a*_ is the initial temperature of the substrate, which was assumed to be at room temperature. The number of pulses *N*_*p*_ can be obtained from the following equation:

2 $${N}_{p}=\frac{f\cdot D}{v}$$

where *D* is the diameter of the laser spot, *f* the repetition rate, and *v* is the scanning speed.

*ΔT*_*rad*_ can be calculated from the analytical solution of the linear heat equation, under the approximation of times much longer than the pulse duration and of a semi-infinite substrate; the substrate properties are also assumed temperature-independent, and the beam is treated as Gaussian. Under these assumptions, the following equation is obtained^26^:

3 $$\Delta {T}_{rad}=\frac{AI{w}_{0}^{2}\tau }{4\sqrt{{}\pi }kt\sqrt{{D}_{PMMA}t}}$$

where *A* is the linear absorption coefficient of PMMA *I* is laser intensity, *w*_*0*_ is the radius of the laser focal spot, *τ* the length of the laser pulse, *D*_*PMMA*_ is the PMMA thermal diffusivity and *k* is its thermal conductivity. *t* is the time between two consecutive pulses (1/*f* ). The absorption coefficient *A* has been directly estimated through transmission measurements and considered temperature independent^22^.

To calculate the contribution to the PMMA heating due to heat conduction from the silicon substrate, i.e., Δ*T*_*cond*_, the Fourier’s law for conduction has been applied to the system, leading to the following equation:

4 $$\Delta {T}_{cond}=\frac{k\left({T}_{Si}-{T}_{a}\right)t}{{d}^{2}{c}_{PMMA}{\rho }_{PMMA}}$$

where *c*_*PMMA*_ is the PMMA specific heat capacity, *ρ*_*PMMA*_ is its density and *d* represent the thickness of PMMA in which the heat propagates. This latter value is set equal to the depth of the modified region of the PMMA which was evaluated from the optical microscope images of the samples and was measured to be around 70 μm. To obtain Δ*T*_*cond*_, however, it is also necessary to calculate the average temperature of the silicon during the bonding process, *T*_*Si*_. The latter value can be calculated applying an established model for heat accumulation, developed by Weber et al.^27^ by extending the single-pulse solutions of the heat conduction equations in laser welding processes^28^ to the case of multiple pulses. Both Weber et al. and Rykalin assume an isotropic, semi-infinite substrate with heat propagation in three dimensions. Applying the model of Weber et al. results in:

5 $${T}_{Si}={T}_{a}+\frac{2\eta {E}_{p}}{{\rho }_{Si}{c}_{Si}\sqrt{{\left(\frac{4\pi {D}_{Si}}{f}\right)}^{3}}}{\sum }_{N=1}^{{N}_{p}}\frac{1}{\sqrt{{N}^{3}}}$$

where *c*_*Si*_ is the silicon specific heat capacity, *ρ*_*Si*_ is its density, and *D*_*Si*_ is its thermal diffusivity.The thermal efficiency *η* in Eq. (5) is set equal to the fraction of radiation transmitted through the PMMA, i.e., *η* = 1*–A*, considering negligible the reflectivity. Despite during the first pulses this cannot be assumed, the first pulses, damaging the silicon, change the reflectivity of the surface. So, for subsequent pulses, the reflectivity can be considered neglectable. Given the high repetition rate, and so the high number of pulses, it is reasonable to disregard the reflectivity of the first pulses and assume that silicon absorbs all the radiation.

With the model now complete, it is possible to use tabulated values, reported in Table 2, to calculate *T*_*PMMA*_ after 24,000 pulses at 800 kHz and 2.0 μJ.

**Table 2:** Parameters values used in the PMMA heating calculation.

Parameter	Value	References	Parameter	Value	References
*ρ*_*Si*_	2330 kg/m^3^	^29^	*ρ*_*PMMA*_	1188 kg/m^3^	^30^
*c*_*Si*_	703 J/kg/K	^29^	*c*_*PMMA*_	1466 J/kg/K	^30^
*D*_*Si*_	8.8·10^–5^ m^2^/s	^29^	*D*_*PMMA*_	1.09·10^–7^ m^2^/s	^31^
*k*	0.193 W/m^2^	^30^	*A*	8.9·10^–4^	^22^

**Figure 9:**
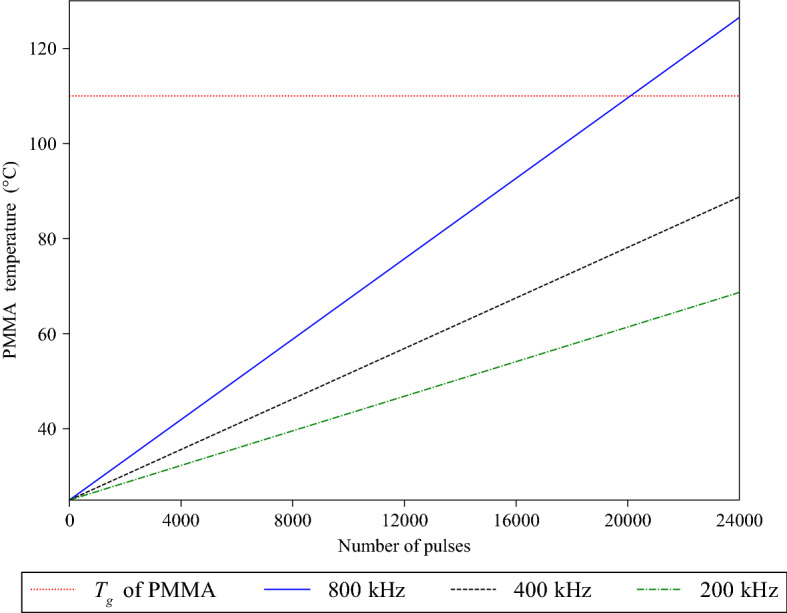
Temperature reached by the PMMA at 200, 400 and 800 kHz after 24,000 pulses. *T*_*g*_ indicates the glass transition temperature of PMMA (110 °C^21^).

Using the values in Table 2, it is obtained that *T*_*PMMA*_ after 24,000 pulses is equal to 126.52 °C. This value is higher than the glass transition temperature of PMMA (110 °C^21^) and as such validates the proposed bonding mechanism. If the contribution of Δ*T*_*cond*_ is neglected, *T*_*PMMA*_ after 24,000 pulses is ~ 40 °C, which is consistent with the observation that PMMA is not modified when irradiated in absence of the silicon substrate. Moreover, if the model is applied again changing the repetition rate value from 800 kHz to 400 and 200 kHz, the PMMA temperature after the same number of pulses is no longer high enough to soften the polymer and create a bonding, as shown in Fig. 9.

## Proof of concept: microfluidic channel sealing and testing

As a feasibility demonstration of femtosecond laser bonding of a hybrid PMMA-Si microfluidic device, we sealed a microfluidic channel previously fabricated by fs-laser ablation on the PMMA substrate, following the same procedure previously developed in^32^. The microchannel had a square cross-section with 400 µm side and a length of 1 cm, and connected two reservoirs, with 3-mm diameter.

After the channel milling, the PMMA sample was flipped upside down and two holes (diameter 1.8 mm) were drilled over the reservoirs to serve as inlet and outlet connections to the external microfluidic network^31^.

**Figure 10:**
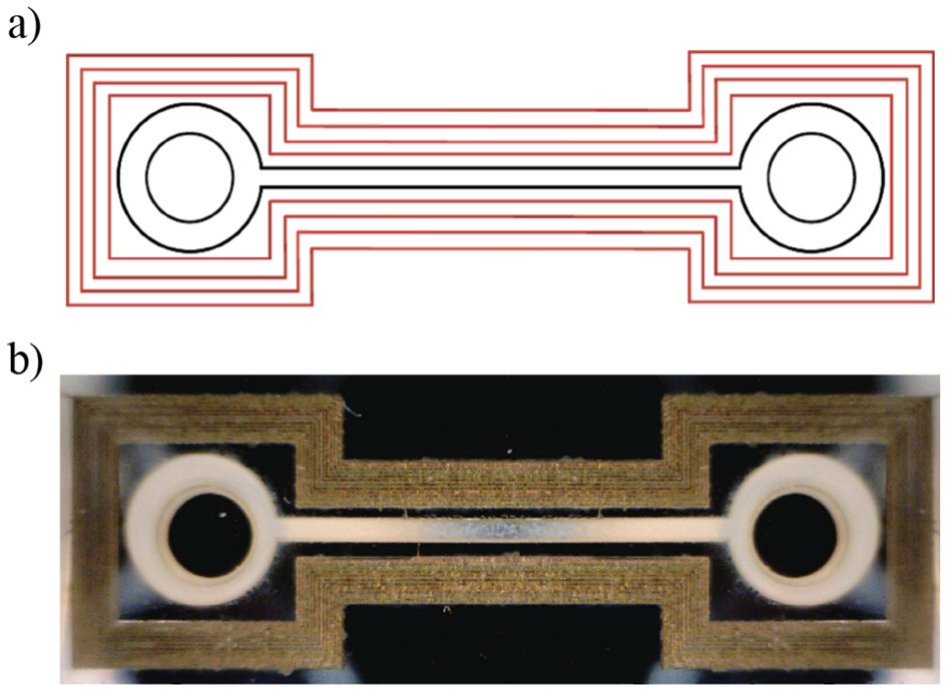
(**a**) Hatch strategy for the bonding with distance between lines 70 µm, and (**b**) corresponding sealed hybrid microfluidic device.

This micro-machined PMMA layer was clamped, and fs-laser bonded to a silicon plate utilizing the process parameters previously found. The laser scanning path was completely surrounding the channel and the reservoirs in order to seal them, as shown in Fig. 10a; in Fig. 10b a picture of the laser bonded device is shown.

To perform the leakage test, one reservoir was connected to the microfluidic pump. Distilled water was injected into the device at increasing pressure up to a value of about 30 mbar, i.e., when a leak was detected. This pressure value corresponds to a flowrate of about 285 μL/min, which is a value almost 30 times higher than the typical operational flow rate of LoCs (1–10 μL/min^33^).

## Conclusions

Bonding of PMMA overlapped onto silicon substrates has been successfully demonstrated by focusing high repetition rate ultrashort laser radiation at the interface between the two layers. Firstly, the influence of repetition rate, scan speed, and pulse energy on the bonding seam has been investigated. A tight window of parameters, where a continue and uniform joining line is obtained, was found. The influence of the hatch distance on the bond strength was also investigated, carrying out shear stress test in push-test configuration. The maximum value of shear stress has been observed when the hatching distance is almost equal to the width of the bonding line. The bonding can be ascribed to the anchoring of the melted PMMA in the laser-generated silicon roughness. In particular, from the thermodynamic model, it emerges that the bonding takes place only when the PMMA is heated beyond its glass transition temperature, which allows it to flow into the trenches ablated into the silicon and then cool down, thus forming mechanical interlocks at the interface between the two materials.

The developed fs-laser based bonding method of PMMA to silicon has been applied to assemble and seal a microfluidic device consisting of a microfluidic channel previously fabricated by fs-laser ablation on the surface of a PMMA slide.

The effectiveness of the sealing has been proved by a dynamic leakage test with a flowrate up to about 285 μL/min, which is an almost 30 times higher value than the typical operational flow rate of LoCs.

The main advantages of the proposed bonding technique are its flexibility, due to the use of a scan head, and the sustainability, being green and chemical free process, with no risk to clog cavities or contaminate samples. Moreover, the proposed technique is unique in its ability to precisely bond very small areas of any desired shape. However, it should be noted that care must be taken in clamping the substrates together in order of efficiently bond them, and that overlapping the bonded area with structures present on the silicon substrate might damage them.

Potential applications of this fs-laser based technique are packaging of integrated photonic devices and assembly of solar battery components, micro-optomechanical systems, microfluidic devices, etc., where full control over size and geometry of the joined area is highly demanded^34^.

## Data Availability

The datasets generated during and/or analyzed during the current study are available from the corresponding author on reasonable request.

## References

[1] Kah P, Suoranta R, Martikainen J, Magnus C (2014). Techniques for joining dissimilar materials: Metals and polymers. Rev. Adv. Mater. Sci..

[2] Fang Y, Jiang X, Mo D, Zhu D, Luo Z (2019). A review on dissimilar metals’ welding methods and mechanisms with interlayer. Int. J. Adv. Manuf. Technol..

[3] Zhang G, Zhu Q, Yang H, Yang C, Liu Y, Wang C (2022). Effect of surface treatments on the laser welding performance of dissimilar materials. J. Manuf. Process..

[4] Çelen S, Efeoǧlu C, Özden H (2011). Pulsed laser-induced micro-pits: As bone stabilizers. Phys. Procedia.

[5] Volpe A, Krishnan U, Chiriacò MS, Primiceri E, Ancona A, Ferrara F (2020). A smart procedure for the femtosecond laser-based fabrication of a polymeric lab-on-a-chip for capturing tumor cell. Engineering.

[6] Temiz Y, Lovchik RD, Kaigala GV, Delamarche E (2015). Lab-on-a-chip devices: How to close and plug the lab?. Microelectron Eng.

[7] Zhang G, Bai J, Zhao W, Zhou K, Cheng G (2017). Interface modification based ultrashort laser microwelding between SiC and fused silica. Opt. Express.

[8] Carter RM (2021). From concept to industry: Ultrafast laser welding. Am. Ceram. Soc. Bull..

[9] Mirza I, Bulgakova NM, Tomáštík J, Michálek V, Haderka O, Fekete L (2016). Ultrashort pulse laser ablation of dielectrics: Thresholds, mechanisms, role of breakdown. Sci. Rep..

[10] Roth GL, Rung S, Hellmann R (2016). Welding of transparent polymers using femtosecond laser. Appl. Phys. A.

[11] Tamaki T, Watanabe W, Nishii J, Itoh K (2002). Welding of transparent materials using femtosecond laser pulses. Jpn. J. Appl. Phys. Part.

[12] Horn A, Mingareev I, Werth A, Kachel M, Brenk U (2008). Investigations on ultrafast welding of glass-glass and glass-silicon. Appl. Phys. A.

[13] Miyamoto I, Okamoto Y, Hansen A, Vihinen J, Amberla T, Kangastupa J (2015). High speed, high strength microwelding of Si/glass using ps-laser pulses. Opt. Express.

[14] Chambonneau M, Li Q, Fedorov VY, Blothe M, Schaarschmidt K, Lorenz M (2021). Taming ultrafast laser filaments for optimized semiconductor-metal welding. Laser Photon Rev..

[15] Sopeña P, Wang A, Mouskeftaras A, Grojo D (2022). Transmission laser welding of similar and dissimilar semiconductor materials. Laser Photon Rev..

[16] Carter RM, Chen J, Shephard JD, Thomson RR, Hand DP (2014). Picosecond laser welding of similar and dissimilar materials. Appl. Opt..

[17] Chen L, Yang G, Wang S (2012). Air-grid surface patterning provided by superhydrophobic surfaces. Small.

[18] Tsao CW, DeVoe DL (2009). Bonding of thermoplastic polymer microfluidics. Microfluid Nanofluid..

[19] Volpe A, Trotta G, Krishnan U, Ancona A (2019). Prediction model of the depth of the femtosecond laser micro-milling of PMMA. Opt. Laser Technol..

[20] Acherjee B, Kuar AS, Mitra S, Misra D, Acharyya S (2012). Experimental investigation on laser transmission welding of PMMA to ABS via response surface modeling. Opt. Laser Technol..

[21] Fernández-Pradas JM, Florian C, Caballero-Lucas F, Morenza JL, Serra P (2013). Femtosecond laser ablation of polymethyl-methacrylate with high focusing control. Appl. Surf. Sci..

[22] Volpe A, di Niso F, Gaudiuso C, de Rosa A, Vázquez RM, Ancona A (2015). Welding of PMMA by a femtosecond fiber laser. Opt. Express.

[23] https://www.microfluidic-chipshop.com/microfluidics/materials-in-microfluidics/polymers-in-microfluidics/pmma/.

[24] Xu F, Liu S, Fan H, Xu Y, Ding Y (2019). Enhancement of the adhesion strength at the metal-plastic interface via the structures formed by laser scanning. Opt. Laser Technol..

[25] Florian C, Fischer D, Freiberg K, Duwe M, Sahre M, Schneider S (2021). Single femtosecond laser-pulse-induced superficial amorphization and re-crystallization of silicon. Materials.

[26] Bäuerle D (2011). Ultrashort-Pulse Laser Ablation. Laser Processing and Chemistry.

[27] Weber R, Graf T, Berger P, Onuseit V, Wiedenmann M, Freitag C (2014). Heat accumulation during pulsed laser materials processing. Opt Express.

[28] Rykalin NN (1957). Die Wärmegrundlagen des Schweißvorgangs.

[29] Silicon Wafer. *American Elements*. https://www.americanelements.com/silicon-wafer-7440-21-3. (2022).

[30] Brandrup J, Immergut EH, Grulke EA (1999). Polymer Handbook.

[31] Rohde M, Hemberger F, Bauer T, Blumm J, Fend T, Häusler T (2013). Intercomparison of thermal diffusivity measurements on CuCrZr and PMMA. High Temp. High Press..

[32] Volpe A, Paiè P, Ancona A, Osellame R (2019). Polymeric fully inertial lab-on-a-chip with enhanced-throughput sorting capabilities. Microfluid Nanofluid..

[33] Delaney C, McCluskey P, Coleman S, Whyte J, Kent N, Diamond D (2017). Precision control of flow rate in microfluidic channels using photoresponsive soft polymer actuators. Lab Chip.

[34] Zoupanou S, Volpe A, Primiceri E, Gaudiuso C, Ancona A, Ferrara F (2021). SMILE platform: An innovative microfluidic approach for on- chip sample manipulation and analysis in oral cancer diagnosis. Microma.

